# Diagnosis and Treatment of Arterial Occlusion after Knee Arthroplasty: The Sooner, the Better

**DOI:** 10.1111/os.12494

**Published:** 2019-06-26

**Authors:** Zeng Li, Shuai Xiang, Yan‐yan Bian, Bin Feng, Rong Zeng, Xi‐sheng Weng

**Affiliations:** ^1^ Department of Orthopedic Surgery Peking Union Medical College Hospital, Peking Union Medical College, Chinese Academy of Medical Science Beijing China; ^2^ Department of Vascular Surgery Peking Union Medical College Hospital, Peking Union Medical College, Chinese Academy of Medical Science Beijing China

**Keywords:** Arterial occlusion, Total knee arthroplasty, Unicondylar knee arthroplasty

## Abstract

Knee arthroplasty, including total knee arthroplasty (TKA) and unicondylar knee arthroplasty (UKA), is an effective procedure for patients with severe knee joint diseases. Arterial occlusion after knee arthroplasty is a rare but severe complication. However, there are few comprehensive reviews or analyses focusing on it. In this study, we presented a case of successful treatment of acute arterial occlusion of the popliteal artery after TKA by emergent balloon angioplasty, and conducted a review and analysis of published cases with this complication. After search and screening, 36 studies with 47 cases of arterial occlusion after knee arthroplasty in the past 35 years (1984–2018) were included. Among the 47 patients, there were 22 men and 25 women. The mean age was 68 years old. A total of 43 patients had primary TKA while 2 had revision surgery for TKA and 2 for UKA. For arterial occlusions, 66% presented symptoms in less than 1 day after knee surgery and 95% of the occlusion sites were around the popliteal artery. For treatment, 89% chose surgical treatment. Compared with conservative treatment, surgical treatment was more effective (*P* < 0.01). The patients who underwent surgical treatment less than 1 day after diagnosis had less sequelae (*P* < 0.05). For arterial occlusion after knee arthroplasty, we should pay attention to the perioperative risk factors and presentations, and diagnose and treat surgically at an early stage.

## Introduction

Knee arthroplasty, including total knee arthroplasty (TKA) and unicondylar knee arthroplasty (UKA), is an effective procedure that can dramatically improve knee function and the quality of life in patients with severe joint diseases. However, arterial occlusion is a rare but severe complication after knee arthroplasty. According to previous studies, the overall incidence of arterial complications after TKA, including arterial occlusion, arteriovenous fistula, arterial aneurysm, and arterial severance, is approximately 0.03% to 0.17%^1^, ^2^. Among these complications, arterial occlusion accounts for approximately 60%^3^. This means that there is less than 1 patient who may suffer from arterial occlusion in 1000 patients who have undertaken knee arthroplasty. Although rare, once it has occurred, it will affect the rehabilitation of the knee joint and may lead to many sequelae, such as chronic pain, drop foot, and the need to walk with aids, which seriously reduce the quality of life of patients. If not found in time or treated correctly, amputation may be performed^4^.

Despite the infrequency of this complication, it is necessary for doctors to have a comprehensive understanding of this problem, including risk factors, presentation, diagnosis, treatment, and prognosis, which can minimize the adverse impacts. Recently, we successfully treated a patient who had arterial occlusion after knee arthroplasty and had a good outcome at 3‐month follow‐up. Therefore, in the present study, we aimed to review the previous published cases and to combine this analysis with our experience to examine and discuss the relative factors relating to this complication.

## Methods

### *Study Selection*

The inclusion criteria were as follows: (i) all participants in the studies must have undergone either TKA or UKA; (ii) patients had arterial occlusions of lower extremities with confirmed diagnosis after knee surgeries; (iii) perioperative details of knee arthroplasty and information of arterial occlusion should be reported; (iv) there were no study design restrictions; and (v) only studies in English were included because of accessibility. The exclusion criteria as follows: (i) patients undergoing arthroplasties of any other joints; (ii) cases without detailed information or confirmed diagnosis; (iii) cases with obvious arterial injury during the knee surgeries.

### *Search Strategy*

**Figure 1:**
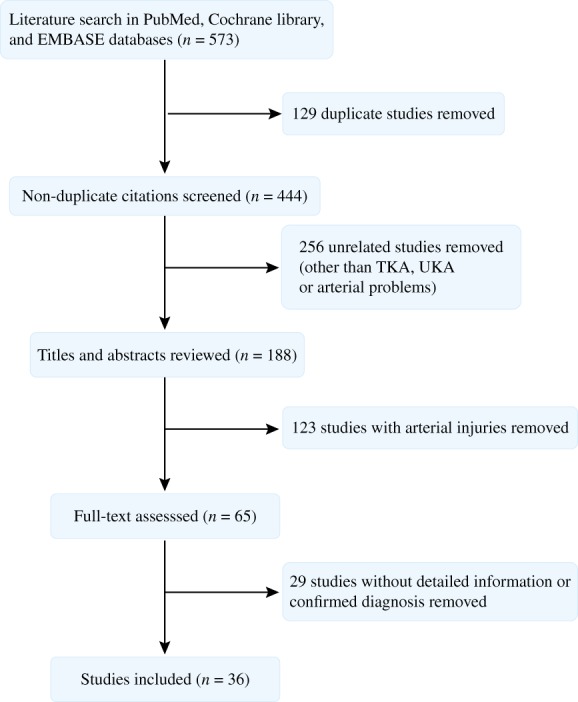
Flow chart demonstrating article selection based on selection criteria.

The literature search was performed on 10 January 2018 through electronic databases: PubMed, Cochrane library, and EMBASE databases. The search terms and Boolean operators used were as follow: (“arthroplasty, replacement, knee” [MeSH Terms] OR “knee arthroplasty” [Free Terms] OR “knee replacement” [Free Terms]) AND ((“arteries” [MeSH Terms] OR “blood vessels” [MeSH Terms] OR “arteries” [Free Terms] OR “artery” [Free Terms] OR “arterial” [Free Terms] OR “vascular” [Free Terms]) AND (“occlusion” [Free Terms] OR “complications” [Subheading] OR “complications” [Free Terms])). No article type restrictions were included in the search strategy. After excluding the duplicates, two reviewers independently screened the studies and included studies based on the criteria (Fig. 1).

### *Data Collection*

Information was extracted by two reviewers independently as follows: first author, year, age, gender, body mass index (BMI), medical history, perioperative details of arthroplasty, and information of arterial occlusion. We attempted to contact the authors by email for the details of cases that were not presented in the papers.

### *Statistical Analysis*

For pooled data, quantitative variables (age, BMI, range of motion, tourniquet time, and tourniquet pressure) were described using mean value and categorical variables (sex, medical history, surgical type, surgical side, presentation, examination, and treatment) were shown as frequency and percentage. The statistical analysis of categorical variables was done by *χ* ^2^ test and Fisher's exact test. Statistical significance was considered when the *P*‐value was less than 0.05. All the analysis was done using SPSS software (version 23.0, Chicago, IL, USA).

## Results

### *Case Presentation*

#### Preoperative Information

**Figure 2:**
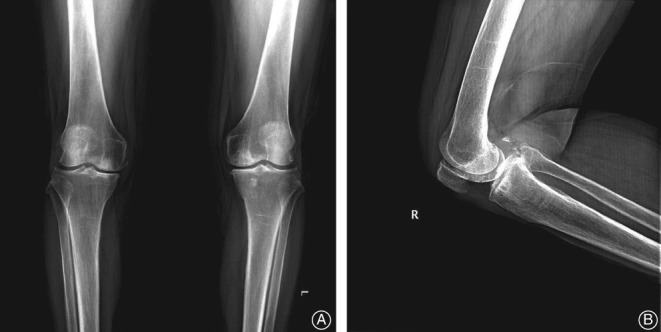
Preoperative X‐ray of the right knee. Preoperative X‐ray showed narrow medial knee joint space and osteophyte formation without vascular calcification. (A) Anteroposterior film. (B) Lateral film.

The patient was a 73‐year‐old Chinese woman with osteoarthritis in both knees. In the recent half year, she had been suffering from intractable right knee pain with failed conservative treatment and prepared for right side TKA. She had a 27‐year history of hypertension and her blood pressure was controlled well by medication. She had no other medical history, including smoking history. Her BMI was 24. The physical examination showed a limited range of motion (ROM) of 0°–120° without varus or valgus deformity. Preoperative X‐ray showed narrow medial knee joint space and osteophyte formation without vascular calcification (Fig. 2). The peripheral pulses and capillary refill were normal, although the preoperative Doppler ultrasound showed arteriosclerosis with plaque formation in the arteries in both legs.

#### Diagnosis and Treatment of Arterial Occlusion

**Figure 3:**
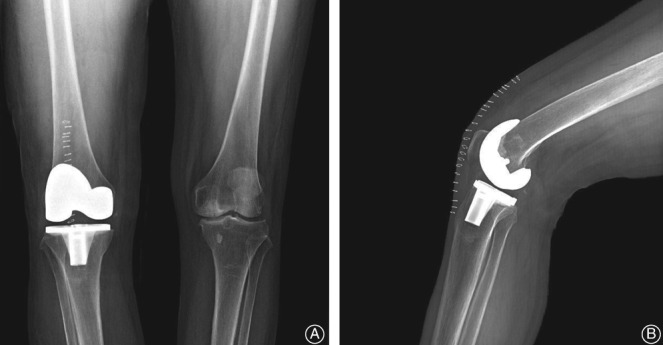
Postoperative X‐ray of the right knee. The implant was installed in the proper position. (A) Anteroposterior film. (B) Lateral film.

The right side TKA with a posterior stabilized design (LPS, NexGen, Zimmer, Warsaw, USA) was performed under general anesthesia. A tourniquet was applied for 70 min at a pressure of 250 mmHg. The whole surgery was completed without any complications and there was normal intraoperative bleeding at the surgical site. The postoperative X‐ray showed successful implantation for the right knee (Fig. 3).

**Figure 4:**
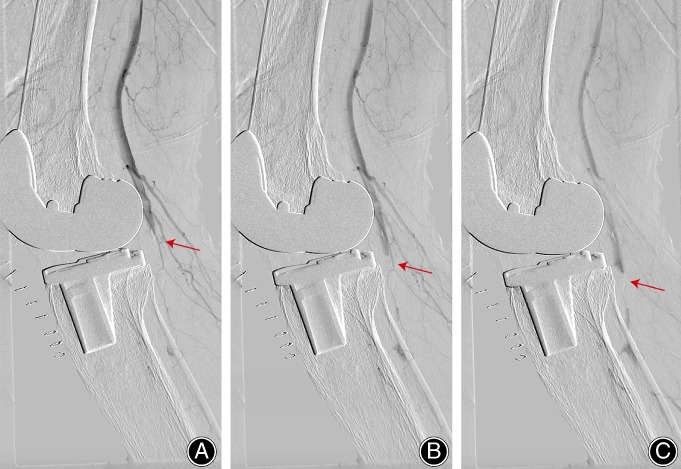
Arteriography prior balloon angioplasty. An emergency arteriography under local anesthesia showed that there was a short segmental occlusion of the popliteal artery. (A–C) Changes over time.

**Figure 5:**
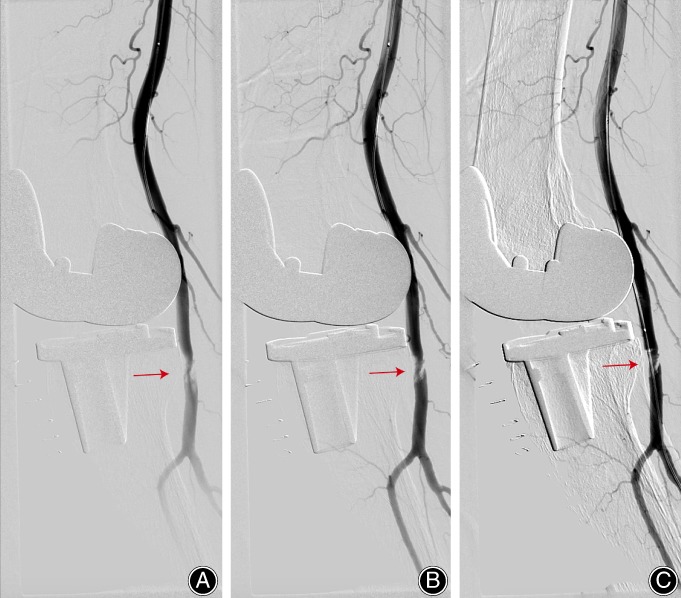
Arteriography after balloon angioplasty. Follow‐up imaging showed excellent blood flow, although vascular stenosis was still observed in the popliteal artery. (A–C) Changes over time.

However, immediately after the surgery, routine peripheral pulse check in the operating room found absent dorsalis pedis artery pulse in the right foot. The patient was closely monitored in the recovery room. After approximately 2 h, there was no improvement in peripheral pulses, capillary refill, and oxygen saturation. In consideration of the high possibility of arterial occlusion, the vascular consultant recommended using 1000 U heparin for anticoagulation and immediate evaluation by arteriography for diagnosis. An emergency arteriography under local anesthesia showed that there was a short segmental occlusion of the popliteal artery (Fig. 4). Then the angioplasty was performed with a balloon with a diameter of 5 mm for 3 min. Follow‐up imaging showed excellent blood flow although vascular stenosis was still observed in popliteal artery (Fig. 5). There was a return of palpable pulses and a normal capillary refill and oxygen saturation.

After the patient returned to the ward, 12 500 U heparin in 50 mL saline was pumped 1 mL/h and activated partial thromboplastin time (APTT) was controlled around 40 s. The peripheral pulses were closely monitored. On postoperative day 2, the patient was allowed to walk with aids. On postoperative day 6, the anticoagulation plan was changed to rivaroxaban 10 mg two times a day and aspirin 100 mg one time a day for 3 weeks orally. The patient was discharged home 2 weeks after surgery without any complaints.

At the most recent follow‐up in the orthopaedic clinic (3 months after surgery), there were no orthopaedic or vascular complaints. The patient had an ROM of 0° to 125° with excellent muscle strength and could walk without aids for 1 km.

### *Pooled Analyses*

#### Characteristics of Studies

After careful search and screening, 36 studies with 47 cases of arterial occlusion after knee arthroplasty in the past 35 years (1984–2018) were included in the analysis (1 from the present study)^1^, ^5^, ^6^, ^7^, ^8^, ^9^, ^10^, ^11^, ^12^, ^13^, ^14^, ^15^, ^16^, ^17^, ^18^, ^19^, ^20^, ^21^, ^22^, ^23^, ^24^, ^25^, ^26^, ^27^, ^28^, ^29^, ^30^, ^31^, ^32^, ^33^, ^34^, ^35^, ^36^, ^37^, ^38^ (Table S1). Demographics, predisposing conditions, perioperative details, arterial occlusion information, and prognosis are shown in Tables 1, 2, 3, 4.

**Table 1:** Demographics and predisposing conditions of arterial occlusion cases after knee arthroplasty

Variables	Number of patients (%)
Age (years)	47
Mean	68.09
<65	17(36.17)
≥65	30 (63.85)
Sex	47
Male	22 (46.81)
Female	25 (53.19)
BMI (kg/m^2^)	7
<25	1 (14.29)
25–30	3 (42.86)
>30	3 (42.86)
Smoker	8 (17.02)
Hypertension	14 (29.79)
Peripheral artery disease	8 (17.02)
Dyslipidemia	5 (10.64)
Diabetes	3 (6.38)
Coronary artery disease	3 (6.38)
Cancer	4 (8.51)
Prostatic cancer	2 (4.26)
Breast cancer	2 (4.26)

BMI, body mass index

**Table 2:** Perioperative details of arterial occlusion cases after knee arthroplasty

Variables	Number of patients (%)
ROM (degree)	23
Mean	89.78
<100	14 (60.87)
≥100	9 (39.13)
Axial deformity	14 (29.79)
Varus	8 (17.02)
Valgus	6 (12.77)
Preoperative peripheral pulses	23
Normal	13 (56.52)
Abnormal	10 (43.48)
Decreased	8 (17.02)
Absent	2 (4.26)
Surgery type	47
Primary TKA	43 (91.49)
Implant type	7
CR	0 (0.00)
PS	5 (71.43)
CCK	1 (14.29)
RH	1 (14.29)
Revision TKA	2 (4.26)
Primary UKA	2 (4.26)
Side	46
Left	19 (41.30)
Right	18 (39.13)
Bilateral	9 (19.57)
Tourniquet	36
Use	32 (88.89)
Time (min)	24
Mean	87.17
<60	6 (25.00)
≥60	18 (75.00)
Pressure (mmHg)	13
Mean	306.54
Abnormal bleeding during surgery	6 (12.77)

CCK, constrained condylar knee; CR, cruciate retaining; PS, posterior stabilizing; RH, rotating hinge; ROM, range of motion; TKA, total knee arthroplasty; UKA, unicondylar knee arthroplasty

**Table 3:** Arterial occlusion information after knee arthroplasty.

Variables	Number of patients (%)
First presentation	44
Pulselessness	16 (36.36)
Poikilotherm	9 (20.45)
Pain	8 (18.18)
Pallor	4 (9.09)
Paresthesia	3 (6.82)
Paralysis	2 (4.55)
Occurring time of first presentation (day)	47
<1	31 (65.96)
>1	16 (34.04)
Imaging examination	47
DSA	38 (80.85)
Doppler US	26 (55.32)
CTA	3 (6.38)
Artery occlusion site	46
Superficial femoral	1 (2.17)
Superficial femoral and femoral‐popliteal junction	1 (2.17)
Superficial femoral and popliteal	5 (10.87)
Popliteal	31 (67.39)
Popliteal and posterior tibial‐peroneal junction	1 (2.17)
Posterior tibial	1 (2.17)
Previous bypass graft or stent	6 (13.04)
Pathology	44
Thrombosis	38 (86.36)
Artery laceration or intimal tear	10 (22.73)
Arteriosclerosis	9 (20.45)
Compression of displaced patellar component	1 (2.27)
Treatment	47
Conservative treatment	5 (10.64)
Surgical treatment	42 (89.36)
Thrombectomy	28 (66.67)
Bypass graft	13 (30.95)
Balloon angioplasty	8 (19.05)
Stent	4 (9.52%)
Amputation	7 (14.89)

CTA, computed tomography angiography; DSA, digital subtraction angiography; US, ultrasound

**Table 4:** Prognosis of arterial occlusion after knee arthroplasty

Variable	Success (*n* †)	Failure‡ (*n* †)	*P*
Age (<65 years)	12 (40)	5 (7)	0.035*
Sex (male)	18 (40)	3 (7)	0.872
Peripheral arterial disease	5 (40)	3 (7)	0.049*
ROM (100°)	12 (20)	2 (3)	0.825
Peripheral pulses, abnormal	6 (19)	4 (4)	0.024*
Tourniquet, no	2 (31)	2 (5)	0.027*
Occlusion, above knee	2 (35)	5 (5)	0.000*
Treatment, surgical	38 (40)	4 (7)	0.003*
Surgical treatment time, <1 day	26 (34)	5 (8)	0.419
Surgical treatment time, <1 day	11 (13)§	4 (9)**	0.047*

* *P* < 0.05.

† Total number.

‡ Recurrence or failure.

§ well.

** Sequelae.

ROM, range of motion.

#### Preoperative Factors

In all 47 patients who suffered arterial occlusions after knee arthroplasty, the mean age was 68 years old and more than 60% patients were older than 65 years old. There were 47% male patients and 53% female patients. BMI was reported for 7 patients, and 6 of them were overweight or obese. The medical history of the patients was mainly focused on hypertension (29%), peripheral arterial diseases (17%), and dyslipidemia (11%). Seventeen percent of the patients were smokers (Table 1). For the knee condition, the mean ROM was 89.78° and in more than 60% was less than 100°. Approximately 30% had varus or valgus deformity (Table 2).

#### Characters of Arterial Occlusions

Most patients had primary TKA (91%), while 2 (4%) had revision surgery for TKA and 2 (4%) for UKA; 80% had unilateral surgery and 20% had bilateral surgery. A tourniquet was used in 89% of cases. From the tourniquet time of 24 cases and tourniquet pressure of 13 cases, the mean time was 87 min and the mean pressure was 307 mmHg. The tourniquet time of 75% cases was more than 60 min (Table 2).

Most arterial occlusions presented less than 1 day after knee surgery (66%) and had symptoms including pulselessness, poikilotherm, pain, paresthesia, pallor, poor capillary refill, paralysis, decreased ankle brachial index (ABI), mottling, poor oxygen saturation, swelling, blister, and necrosis. The most common presentation was pulselessness (84%). For diagnosis, the most common and effective imaging examination was digital subtraction angiography (DSA, 81%) and 95% of the occlusion sites were around the popliteal artery (excluding occlusions in previous grafts or stents). For pathology, 86% cases were confirmed to be arterial thrombosis after imaging examination or surgery (Table 3).

#### Treatments of Arterial Occlusions

There were various treatments for arterial occlusion. The conservative treatment included anticoagulation, thrombolysis, vasodilation, and fasciotomy, while surgical treatment included thrombectomy, balloon angioplasty, bypass graft, and stent. Most patients chose surgical treatment (89%). The most common surgical treatment was surgical or endovascular thrombectomy (67%) (Table 3). Failure was defined as amputation, including knee disarculation, below‐knee amputation or above‐knee amputation (Table 4). Compared with the conservative treatment, surgical treatment had a much higher success rate (90% to 40%, *P* < 0.01). For the prognosis of arterial occlusion after knee arthroplasty, age younger than 65, arterial disease history, abnormal preoperative peripheral pulses, no tourniquet use, and artery occlusion above the popliteal level were associated with higher risk of failure of arterial occlusions treatment after knee arthroplasty (*P* < 0.05). Although the interval between presentation and surgical treatment (less or more than 1 day) did not affect the success rate for the arterial occlusions (*P* > 0.05), the interval less than 1 day showed less sequelae in a mean follow up of 16 months (*P* < 0.05) (Table 4).

## Discussion

In the present study, we collected detailed information for 47 cases presenting over the past 35 years. This is now the largest review and analysis of this rare complication (Table S1).

From the baseline data of the patients with arterial occlusion after knee arthroplasty, people with age older than 65 years old, BMI more than 25, history of hypertension, or peripheral arterial diseases comprised a large proportion in this group. On the other hand, dyslipidemia, diabetes, and smoking were also on the list (Table 1). In fact, all these factors indicated poor condition of arteries which were prone to occlusion. Interestingly, we also found that there were 4 patients who had a history of cancer, 2 for prostatic cancer and 2 for breast cancer (Table 1). The cancer history might lead to a hypercoagulable state so that thrombosis is formed more easily than in others^39^, ^40^. Therefore, these people should be considered as having high risk of arterial occlusion after knee arthroplasty.

Before the surgery, physical examination is necessary. A preoperative peripheral pulses test is an important one. In 23 cases of arterial occlusion, 43% of patients had decreased or absent pulses (Table 2). In our experience, doctors could draw markers on both sides of dorsalis pedis arteries before the surgery so that pulses can be checked and compared with the opposite side more easily. In addition, we recommend checking the ABI, capillary refill, and oxygen saturation routinely. For the patients with high risk, Doppler ultrasound examination could provide more precise judgment on the arterial condition.

For TKA, implant design might have some influence on the artery occlusion. In the 7 cases which reported the implant types, none of them were the cruciate retaining design (CR, 0), which needed to protect the posterior cruciate ligament in the surgery and had a low possibility of injury to the posterior artery. In contrast, constrained prosthesis designs such as the posterior stabilizing design (PS, 5), the constrained condylar knee design (CCK, 1) and the rotating hinge design (RH, 1) might have a higher risk compared with CR, especially for knees with severe deformity which need further osteotomy or soft tissue release, such as posterior cruciate ligament or posterior capsule injuries (Table 2). In the preoperative physical examination, the ROM of 61% patients was less than 100° and 30% were varus or valgus deformity (Table 2). Therefore, full preparation before surgery and careful performance during surgery is necessary. Tourniquet is another important factor. Most occlusion cases had experience of tourniquet use and duration of more than 60 min (Table 2). When releasing the tourniquet, doctors should pay attention to the bleeding; unusual slight or significant bleeding always indicates occlusion or injury of the artery, which may lead to occlusion. There were 6 cases in which abnormal bleeding was reported during the surgery.

As the most common presentation, pulselessness always occurs within 1 day after surgery, so pulses should be checked immediately after surgery and monitored regularly in the recovery room and wards for several days, especially for high‐risk patients. If the pulses are abnormal, attention should be paid to other symptoms, including ABI, capillary refill, and oxygen saturation; Doppler ultrasound examination should be used to exclude the possibility of arterial occlusion.

The first presentation as a warning of occlusion is very important and useful to doctors. We could generalize symptoms into 6P, including pulselessness (36%), poikilotherm (20%), pain (18%), pallor (9%), paresthesia (7%), and paralysis (5%) (Table 3). Once arterial occlusion is highly suspicious, DSA should be taken to make a diagnosis in time so that it will save time for the doctors to undertake further treatment. Compared with conservative treatment, surgical treatment was more effective. Although a timely surgical treatment may not affect the successful rate of artery recanalization, it could play an important role in reduction of sequelae in the future. For treatment, age younger than 65 years old, arterial disease history, abnormal preoperative peripheral pulses, no tourniquet use, and artery occlusion above the popliteal level were associated with a lower success rate, which doctors should take into consideration. (Table 4). Among these factors, age younger than 65 years and no tourniquet use seem paradoxical because they are not risk factors of arterial occlusion. In fact, if a patient who is young or as a knee surgery without tourniquet still experiences arterial occlusion, this may indicate a high possibility of severe iatrogenic arterial injury, which always leads to a bad result.

Arterial occlusion is a rare but severe complication of knee arthroplasty. However, if we pay attention to the perioperative risk factors and presentations, and diagnose and treat patients surgically at an early stage, it may not influence the quality of life of patients in the future. In brief, the time of diagnosis and treatment is crucial for arterial occlusion after knee arthroplasty.

## Supporting information


**Table S1:** Characters of the included studies and cases
