# Supplementary material for: Diagnosis and Treatment of Arterial Occlusion after Knee Arthroplasty: The Sooner, the Better
Source: Orthop Surg. 2019 Jun 26;11(3):366–72. doi: 10.1111/os.12494 (PMC6595109; doi:10.1111/os.12494)
Supplement: Supplementary file 1 — Table S1: Characters of the included studies and cases [file OS-11-366-s001.pdf]

Supplement

Table S1. Characters of the included studies and cases

Author	Year	Age(Y)	Sex	Surgery	Side
McAuley [5]	1984	60	M	Primary TKA	L
McAuley [5]	1984	62	M	Primary TKA	Bi
Rand [1]	1987	78	M	Primary TKA	Bi
Rand [1]	1987	86	F	Primary TKA	R
Rand [1]	1987	64	F	Primary TKA	L
Rush [6]	1987	58	M	Primary TKA	R
Zahrani [7]	1989	79	M	Primary TKA	Bi
Zahrani [7]	1989	62	M	Primary TKA	R
Hagan [8]	1990	80	M	Primary TKA	R
Parfenchuck [9]	1994	74	M	Primary TKA	L
Holmberg [10]	1996	72	F	Primary TKA	Bi
Holmberg [10]	1996	74	F	Primary UKA	R
Mureebe [11]	1996	71	M	Primary TKA	R
Mureebe [11]	1996	83	F	Primary TKA	L
Ohira [12]	1997	71	M	Primary TKA	L
Kumar [13]	1998	76	M	Primary TKA	R
Bellemans [14]	1999	70	M	Primary TKA	L
Bellemans [14]	1999	52	M	Primary TKA	R
Kobayashi [15]	1999	72	M	Primary TKA	R
Gerrand [16]	2000	19	M	Primary TKA	L
Turner [17]	2001	64	F	Primary TKA	L
Turner [17]	2001	83	M	Primary TKA	L
Berger [18]	2002	72	M	Primary TKA	R
Dossche [19]	2002	83	F	Primary TKA	R
Matziolis [20]	2004	69	F	Primary TKA	R
Matziolis [20]	2004	74	F	Primary TKA	L
Shabat [21]	2004	73	F	Primary TKA	L
Gregory [22]	2006	81	F	Primary TKA	-
Shapiro [23]	2006	76	F	Primary TKA	Bi

Kort [24]	2007	44	F	Primary UKA	L
Bayne [25]	2008	55	M	Primary TKA	Bi
Sedrick [26]	2009	60	F	Primary TKA	L
Tangsataphorn [27]	2009	62	F	Primary TKA	Bi
Pal [28]	2010	71	M	Primary TKA	R
Pal [28]	2010	69	M	Primary TKA	R
Pant [29]	2011	60	F	Primary TKA	Bi
Kovacs [30]	2012	67	F	Primary TKA	L
Kovacs [30]	2012	45	F	Revision TKA	R
Mathew [31]	2014	54	F	Primary TKA	R
Chikkanna [32]	2015	54	F	Primary TKA	L
Kehagias [33]	2015	78	F	Primary TKA	L
Papadopoulos [34]	2015	76	F	Primary TKA	L
Tsujimoto [35]	2015	83	M	Revision TKA	R
He [36]	2016	64	F	Primary TKA	L
Inomata [37]	2017	80	F	Primary TKA	Bi
Reynolds [38]	2017	72	M	Primary TKA	L
Present study	2018	73	F	Primary TKA	R

Y, year; M, male; F, female; L, left; R, right; Bi: bilateral; TKA, total knee arthroplasty; UKA, unicondylar knee arthroplasty.
